# Effect of rejection sensitivity on the development of anxious-depressive attack in Japanese outpatients: The mediating roles of rumination, social anxiety, and depressive symptoms

**DOI:** 10.3389/fpsyg.2022.1016879

**Published:** 2022-11-30

**Authors:** Shota Noda, Mina Masaki, Tomokazu Kishimoto, Hisanobu Kaiya

**Affiliations:** ^1^Panic Disorder Research Center, Warakukai Medical Corporation, Tokyo, Japan; ^2^Tokyo Mindfulness Center, Tokyo, Japan; ^3^Nagoya Mental Clinic, Warakukai Medical Corporation, Aichi, Japan; ^4^Department of Psychiatry, Kyoto Prefectural University of Medicine, Kyoto, Japan

**Keywords:** anxious-depressive attack, rejection sensitivity, rumination, social anxiety symptoms, depressive symptoms

## Abstract

**Objective:**

Anxious-depressive attack (ADA) is a cluster of symptoms, including sudden and intense anxiety or depression, intrusive rumination about negative memories or future worries, prominent agitation, impatient behavior, and/or loneliness; in some cases, symptoms include a wide range of violent coping behaviors to manage emotional distress. Four characteristics—rejection sensitivity, rumination, social anxiety symptoms, and depressive symptoms—are thought to be associated with the development of ADA. However, the complex relationships among these factors have not been clarified. In this study, we aimed to examine the mechanism by which these four characteristics influence the development of ADA.

**Methods:**

We conducted a structured interview about ADA with 332 outpatients, who completed several self-report measures, to assess rejection sensitivity, rumination, social anxiety symptoms, and depressive symptoms.

**Results:**

A structural equation model showed goodness-of-fit with the data. These findings suggest that rejection sensitivity may demonstrate a direct effect on the occurrence of ADA. Furthermore, rejection sensitivity might affect depressive symptoms through rumination and social anxiety symptoms and consequently contribute to the development of ADA.

**Conclusion:**

These results provide preliminary evidence that rejection sensitivity contributes to the development of ADA.

## Introduction

Anxious-depressive attack (ADA) is a complex of four symptoms: (1) abrupt outbursts of anxious and/or depressive feelings; (2) intrusive rumination about painful memories or future worries with or without flashbacks; (3) prominent agitation, unrest, or loneliness; and, in some cases, (4) a wide range of coping behaviors to manage emotional distress (Kaiya, 2017). Table 1 presents the diagnostic criteria of ADA (Kaiya et al., 2020, revised). The prevalence of ADA among new patients visiting clinics for mood and anxiety disorders was estimated to be 16.88% (Matsumoto et al., 2020). ADA is a trans-diagnostic symptom complex, and many affected patients demonstrate comorbid mood and anxiety disorders, such as major depressive episodes, agoraphobia, social anxiety disorder (SAD), generalized anxiety disorder, and panic disorder (Kaiya, 2016; Noda et al., 2021). Patients with severe ADA symptoms tend to exhibit severe anxiety and depressive symptoms (Kaiya, 2017; Noda et al., 2021). In many affected patients, ADA causes moderate to severe disturbances to activities of daily living (Noda et al., 2021).

**Table 1:** Diagnostic criteria for anxious-depressive attack.

A. Anxious-depressive attack occurs suddenly and recurrently regardless of one’s situation in various mental disorders.
B. The following symptoms occur in descending order of frequency, but symptom no. 4 is not always present:
1. Abrupt surge of intense discomfort, consisting of mixed emotions of an anxious and depressive nature, with or without the urge to weep. The discomfort peaks within several seconds or less than a minute after onset.
2. Intrusive rumination, including mostly negative memories, consisting of mainly recent or past adverse events (flashbacks) or, in rare cases, worry, which continues for a range of time from several minutes to several hours.
3. Prominent agitation, unrest, or loneliness that occurs during rumination and is occasionally very violent and inappropriate in comparison to ruminative subjects.
4. Various coping behaviors to manage intense discomfort occasionally appear.
C. Physical symptoms (e.g., shortness of breath and palpitations) are extremely mild.
D. The disturbance is not attributable to the direct psychological effects of any stress, physiological effects of a substance, or a neurological or other medical condition.
E. The disturbance is not better explained by another neuropsychiatric disorder (e.g., panic disorder, post-traumatic stress disorder, nonepileptic seizure, frontal epilepsy, intermittent explosive disorder, anxious distress specified for depression, sudden emotional excitement of schizophrenia, or *Ataque de nervios*).

Adapted from Kaiya et al. (2020), with permission from The Japanese Society of Anxiety and Related Disorders (JSARD).

Results of a previous study indicated that rejection sensitivity plays a role in the development of ADA in patients with SAD in the following three ways: (1) rejection sensitivity triggers ADA directly; (2) rejection sensitivity intensifies depressive symptoms, which leads to ADA; and (3) rejection sensitivity intensifies social anxiety symptoms, which worsens depressive symptoms, which in turn leads to ADA (Kaiya et al., 2020). That study showed that rejection sensitivity is of central importance in the occurrence of ADA.

Rejection sensitivity refers to a disposition toward anxious anticipation, ready perception, and overreaction to rejection (Downey and Feldman, 1996). Rejection sensitivity is a criterion specifying atypical features in bipolar and related disorders and depressive disorders as per the Diagnostic and Statistical Manual of Mental Disorders, Fifth Edition (DSM-5; American Psychiatric Association, 2013). Rejection sensitivity is also one of the core characteristics of SAD (Liebowitz, 1987; Harb et al., 2002), and leads to clinically troublesome pathological states or conditions, such as suicidal ideation (Brown et al., 2019), aggression and victimization (Gao et al., 2021), social–emotional maladjustment (Godleski et al., 2019), and low self-esteem and loneliness (Zhou et al., 2020). Rejection sensitivity is associated with anxiety, depressive symptoms, loneliness, borderline personality disorder, and body dysmorphic disorder (Gao et al., 2017) and is thought to be caused by family conflict and maternal harshness in both childhood and adulthood (Godleski et al., 2019).

Rejection sensitivity increases rumination (Pearson et al., 2011), which is characterized by obsessional thinking involving excessive, repetitive thoughts of themes that interfere with other forms of mental activity (American Psychological Association, n.d.). Nolen-Hoeksema et al. (2008) demonstrated that rumination predicts the onset of depression, exacerbates depression, enhances negative thinking, impairs problem-solving skills, interferes with instrumental behavior, and erodes social support. Rumination may also be an important sign of severe clinical symptoms, including depression, dysphoria, suicidal ideation, cognitive complaints, post-traumatic stress symptoms, and aggression, in patients with a history of childhood adversities (Mansueto et al., 2021). Furthermore, rumination is associated with several psychological symptoms, including eating disorders (Palmieri et al., 2021) and emotion regulation difficulties (Mansueto et al., 2022). According to Pearson et al. (2011), rumination correlates with a specific maladaptive interpersonal style consisting of submissive (overly accommodating, nonassertive, and self-sacrificing) behaviors characteristic of SAD. Individuals with SAD or major depressive disorder reported higher levels of rumination than healthy controls, with no difference in the levels of rumination between the two disorders. Moreover, the comorbidity of these two disorders is prominently associated with rumination (Arditte Hall et al., 2019). The intensity of social anxiety correlates with rumination over time in patients undergoing cognitive–behavioral therapy for SAD (Brozovich et al., 2015).

SAD is closely related to depression. A national epidemiologic survey on alcohol and related conditions revealed that the lifetime prevalence of any mood disorder among patients with SAD was 56.3% (Grant et al., 2005). In Japan, SAD is a strong predictor of the first onset of depression (Tsuchiya et al., 2009). Compared with SAD alone, the combination of SAD and depression exhibits a more malignant course characterized by an increased risk of suicide attempts and disease chronicity (Stein, 2006). Individuals with depression comorbid with anxiety disorders, especially SAD, often exhibit atypical features. Koyuncu et al. (2015) reported that 77.1% of depression comorbid with SAD was atypical.

**Figure 1:**
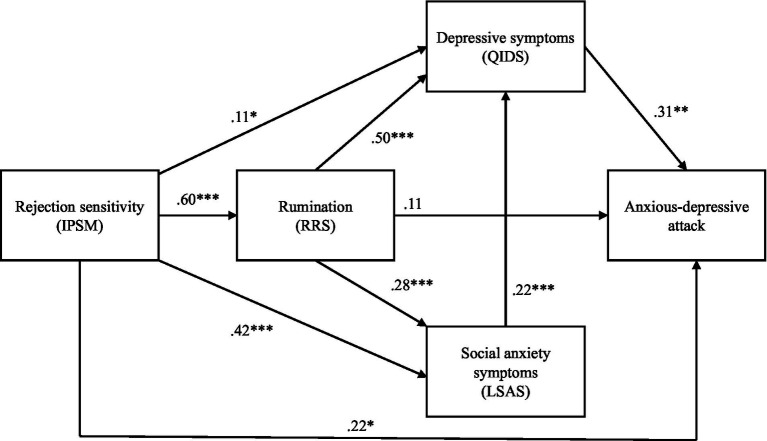
The structural equation model, showing the relationships among rejection sensitivity, rumination, social anxiety symptoms, depressive symptoms, and anxious-depressive symptoms. Chi-square value: *χ*^2^ = 0.10, *df* = 1, *p* = 0.76; comparative fit index = 1.000; Tucker–Lewis Index = 1.000; root mean square error of approximation = 0.000, 90% confidence interval = 0.000–0.099; and standardized root mean square residual = 0.004. IPSM, Interpersonal Sensitivity Measure; RRS, Ruminative Responses Scale; LSAS, Liebowitz Social Anxiety Scale; QIDS, Quick Inventory of Depressive Symptomatology. *p < 0.05; **p < 0.01; ***p < 0.001.

Based on the information above, rejection sensitivity, rumination, social anxiety symptoms, and depressive symptoms, especially with atypical features, have mutual relationships and may contribute to the pathogenesis of ADA. Thus, to reanalyze the pathogenesis of ADA using structural equation modeling (SEM), in the current study, we added rumination as a causative element in patients with SAD and in other patients who visited anxiety-depression clinics. We hypothesized that rumination, social anxiety symptoms, and depressive symptoms mediate the relationship between rejection sensitivity and ADA. To examine the relationship among these variables, we constructed a hypothetical model (Figure 1) and examined the validity of the model.

## Materials and methods

### Participants

Participants were outpatients seeking treatment at a clinic for mood and anxiety disorders in Nagoya/Aichi Prefecture, Japan, aged ≥18 years. Exclusion criteria included high suicide risk, severe physical illness, and significant cognitive impairment. After we obtained written informed consent, 421 outpatients participated in the survey. Of these, 332 completed all the scales and participated in the interview. To examine the effect of rejection sensitivity on the development of ADA, we analyzed the data of these 332 participants.

### Diagnosis and measures

Mental illness was diagnosed in accordance with the DSM-5 (American Psychiatric Association, 2013). In addition, ADA was diagnosed through a structured interview with the participants using the diagnostic criteria for ADA (Kaiya, 2017). ADA was regarded as present only when the diagnoses of one of the two psychiatrists (Tomokazu Kishimoto and Hisanobu Kaiya) and a psychologist (Mina Masaki) were in agreement. We used the following self-administered measures to assess patients’ symptoms:

#### Japanese version of the interpersonal sensitivity measure

The interpersonal sensitivity measure (IPSM) was developed by Boyce and Parker (1989) to assess the tendency to be sensitive to rejection by others. Each item is scored from 1 (very unlike you) to 4 (very like you). Suyama et al. (2014) developed the Japanese version of the IPSM, which consists of five subscales, including “fear of breakup of a relationship,” “unassertive interpersonal behavior due to fear of hurting others,” “fear of criticism by others,” “discrepancy between social self-image and true self-image,” and “obsequence to others.” The scale has 27 items, and the total score ranges from 27 to 108, with higher scores indicating greater sensitivity to rejection by others. The Japanese version of the IPSM has good internal consistency and convergent validity (Suyama et al., 2014) and exhibited good internal consistency in this study (Cronbach’s *α* = 0.92).

#### Japanese version of the ruminative response scale

The ruminative response scale (RRS) was developed by Treynor et al. (2003) to assess rumination about brooding, reflection, and depressive symptoms. The scale consists of 22 items, and each item is scored from 1 (almost never) to 4 (almost always). The total score ranges from 22 to 88, with higher scores indicating greater rumination. The Japanese version of the RRS has good internal consistency, test–retest reliability, and convergent validity (Hasegawa, 2013) and exhibited good internal consistency in this study (Cronbach’s *α* = 0.94).

#### Japanese version of the Liebowitz social anxiety scale

The Liebowitz social anxiety scale (LSAS) was developed by Liebowitz (1987) to assess social anxiety symptoms reflected by anxiety and avoidance of social situations. The scale consists of 24 items each for anxiety and avoidance behavior. The sum of these items is the score for social anxiety symptoms. Each item is rated on a 4-point scale from 0 (none on the anxiety scale and never on the avoidance behavior scale) to 3 (severe on the anxiety scale and usually on the avoidance behavior scale). The total score ranges from 0 to 144, with higher scores indicating greater social anxiety symptoms. The Japanese version of the LSAS has good internal consistency, test–retest reliability, factorial validity, and convergent validity (Asakura et al., 2002; Okajima et al., 2007) and exhibited good internal consistency in this study (Cronbach’s *α* = 0.98).

#### Japanese version of the quick inventory of depressive symptomatology

The quick inventory of depressive symptomatology (QIDS) was developed by Rush et al. (2003) to assess depressive symptoms according to nine symptom domains in major depressive disorder, including sleep and appetite/weight change. Items are scored from 0 to 3. The total score ranges from 0 to 27, with higher scores indicating greater depressive symptoms. The Japanese version of the QIDS has good internal consistency and convergent validity (Fujisawa et al., 2010) and exhibited good internal consistency in this study (Cronbach’s *α* = 0.84).

### Statistical analyses

First, chi-square and *t*-tests were used to compare patients with and without ADA. Second, using weighted least-squares mean-and variance-adjusted estimators, SEM was performed to determine how rumination, social anxiety symptoms, and depressive symptoms mediate the association between rejection sensitivity and ADA. The Statistical Package for the Social Sciences version 25 (IBM Corp., Armonk, NY, the United States) was used to conduct the chi-square and *t*-tests, and SEM was performed using Mplus 8 (Muthén and Muthén, 1998–2017).

### Ethical considerations

This study was approved by the Medical Corporation Warakukai Ethics Review Board. Written informed consent was obtained from all participants prior to their enrollment in the study.

## Results

### Demographics of patients

Table 2 lists psychiatrists’ diagnoses and the clinical characteristics of the participants. Of the 332 patients, 141 were men, and 191 were women (mean age, 34.35 ± 12.92 years). Depressive disorders were diagnosed in 103 patients (31.02%) and anxiety disorders in 97 (29.22%). The presence of ADA was confirmed in 59 patients (12 men and 47 women; mean age, 27.73 ± 7.15 years) and the absence of ADA in 273 patients (129 men and 144 women; mean age, 35.78 ± 13.45 years). Thus, the prevalence of ADA was 17.77%. Furthermore, the patient group with ADA consisted of more females and younger patients than the group of patients without ADA (*p* < 0.01). Patients with ADA demonstrated high rates of comorbidity, including major depressive disorder (33.90%), major depressive disorder with atypical features (28.81%), SAD (8.47%), panic disorder (6.78%), and panic disorder with agoraphobia (5.08%). The rate of comorbid major depressive disorder with atypical features was significantly higher in patients with ADA compared with the rate in patients without ADA (*p* < 0.01).

**Table 2:** Clinical characteristics of participants.

Demographics	All patients (*N* = 332)	Patients with ADA (*n* = 59)	Patients without ADA (*n* = 273)	Chi-square value/*t*-value
Number of men	141 (100%)	12 (8.51%)	129 (91.49%)	14.38**
Mean age (*SD*)	34.35 (±12.92)	27.73 (±7.15)	35.78 (±13.45)	6.51**
Primary diagnosis, *n*				
Autism spectrum disorder	3 (100%)	0 (0.00%)	3 (100%)	0.65
Attention-deficit/hyperactivity disorder	14 (100%)	0 (0.00%)	14 (100%)	3.16
Delusional disorder	1 (100%)	0 (0.00%)	1 (100%)	0.22
Schizophrenia^†^	3 (100%)	1 (33.33%)	2 (66.67%)	0.50
Bipolar disorder^†^	6 (100%)	1 (16.67%)	5 (83.33%)	0.01
Major depressive disorder^†^	80 (100%)	20 (25.00%)	60 (75.00%)	3.77
Major depressive disorder with atypical features^†^	19 (100%)	17 (89.47%)	2 (10.53%)	70.91**
Major depressive disorder with a seasonal pattern	2 (100%)	0 (0.00%)	2 (100%)	0.44
Persistent depressive disorder	2 (100%)	0 (0.00%)	2 (100%)	0.44
Specific phobia^†^	8 (100%)	1 (12.50%)	7 (87.50%)	0.16
Social anxiety disorder^†^	37 (100%)	5 (13.51%)	32 (86.49%)	0.52
Panic disorder^†^	29 (100%)	4 (13.79%)	25 (86.21%)	0.34
Agoraphobia^†^	1 (100%)	1 (100%)	0 (0.00%)	4.64
Panic disorder with agoraphobia^†^	18 (100%)	3 (16.67%)	15 (83.33%)	0.02
Generalized anxiety disorder	4 (100%)	0 (0.00%)	4 (100%)	0.88
Obsessive–compulsive disorder^†^	18 (100%)	2 (11.11%)	16 (88.89%)	0.58
Post-traumatic stress disorder	3 (100%)	0 (0.00%)	3 (100%)	0.65
Acute stress disorder	1 (100%)	0 (0.00%)	1 (100%)	0.22
Adjustment disorder	17 (100%)	0 (0.00%)	17 (100%)	3.87
Insomnia disorder^†^	15 (100%)	1 (6.67%)	14 (93.33%)	1.33
Narcolepsy	1 (100%)	0 (0.00%)	1 (100%)	0.22
Unspecified personality disorder	1 (100%)	0 (0.00%)	1 (100%)	0.22
Depressive state^†^	14 (100%)	2 (14.29%)	12 (85.71%)	0.12
Other (unspecified or other symptoms)^†^	35 (100%)	1 (2.86%)	34 (97.14%)	5.96*
Descriptive statistics				
Interpersonal sensitivity measure	77.52 (±14.08)	86.27 (±10.73)	75.63 (±14.02)	−6.41**
Ruminative responses scale	53.52 (±15.65)	63.46 (±11.96)	51.38 (±15.54)	−6.64**
Quick inventory of depressive symptomatology	11.97 (±5.80)	16.08 (±4.58)	11.08 (±5.65)	−6.37**
Liebowitz social anxiety scale	48.73 (±35.23)	68.49 (±34.32)	44.46 (±34.00)	−4.91**

ADA, anxious-depressive attack; SD, standard deviation; ^†^Includes patients with ADA; *p < 0.05; **p < 0.01.

### SEM

The results of the hypothesized model showed goodness-of-fit with the data (*χ*^2^ = 0.10, *df* = 1, *p* = 0.76; comparative fit index = 1.000; Tucker–Lewis Index = 1.000; root mean square error of approximation = 0.000, 90% confidence interval = 0.000–0.099; and standardized root mean square residual = 0.004). The standardized parameter estimates for rejection sensitivity were significant with regard to rumination (*β* = 0.60, *p* < 0.001), social anxiety symptoms (*β* = 0.42, *p* < 0.001), depressive symptoms (*β* = 0.11, *p* < 0.05), and ADA (*β* = 0.22, *p* < 0.05). The estimates for rumination were significant with regard to social anxiety symptoms (*β* = 0.28, *p* < 0.001) and depressive symptoms (*β* = 0.50, *p* < 0.001). In contrast, the estimate for rumination with regard to ADA was not significant (*β* = 0.11, *p* = 0.40). Furthermore, the estimate for social anxiety symptoms with regard to depressive symptoms was significant (*β* = 0.22, *p* < 0.001), as was the estimate for depressive symptoms with regard to ADA (*β* = 0.31, *p* < 0.01). The model is shown in Figure 1.

## Discussion

The present study aimed to determine the association between rejection sensitivity and rumination, social anxiety symptoms, depressive symptoms, and ADA in Japanese outpatients who visited a mood and anxiety disorder clinic. We hypothesized that rumination, social anxiety symptoms, and depressive symptoms demonstrate significant mediating effects on the relationship between rejection sensitivity and ADA, and we used SEM to test this hypothesis. The results of SEM showed that the hypothesized model’s goodness-of-fit indices demonstrated high values. Our SEM model matched substantially, which can be explained by the two reasons. Firstly, our previous research (Kaiya et al., 2020) has established a model similar to the present model, except for the item of ruminative thinking. Secondly, as mentioned in the introduction, a close relationship between the variables in our SEM model has been clinically demonstrated. This suggested that (1) rejection sensitivity is directly associated with ADA, (2) depressive symptoms mediate the relationship between rejection sensitivity and ADA, and (3) social anxiety symptoms mediate the relationship between rejection sensitivity and depressive symptoms; all these findings are consistent with those in a previous study (Kaiya et al., 2020). In addition, rumination was shown to mediate the relationship between rejection sensitivity and social anxiety or depressive symptoms. The relationship between rejection sensitivity and rumination was significant, but that between rumination and ADA was not. Therefore, we consider that rumination is not directly associated with ADA but is associated with depressive symptoms that trigger ADA. These findings suggest that depressive symptoms may mediate the effect of rejection sensitivity on the development of ADA and that rumination and social anxiety symptoms may mediate the effect of rejection sensitivity on depressive symptoms that trigger ADA.

In the present study, the prevalence rates of ADA in all subjects and in patients with atypical depression were 17.77 and 89.54%, respectively, which were compatible with those of our previous reports (Kaiya, 2017; Matsumoto et al., 2020). The finding that ADA was most common in atypical depression is reasonable because rejection sensitivity, which is considered one of the basic psychopathological manifestations of ADA, as shown in this study, was reported as a symptom of high diagnostic significance in atypical depression (Thase, 2009).

The severity of ADA symptoms is positively associated with anxiety and depressive symptoms, and ADA symptoms disrupt the lives of many affected patients (Noda et al., 2021). Therefore, in patients with anxiety and depressive symptoms, ADA should be identified as early as possible. In this study, rejection sensitivity and depressive symptoms were directly related to ADA, which suggests that patients with high levels of rejection sensitivity and depressive symptoms are at a high risk for developing ADA.

Furthermore, this model suggests that rejection sensitivity is an important predictor of ADA. Therefore, interventions targeting rejection sensitivity should be implemented in the management and prevention of ADA. Mindfulness training may be an option for treating ADA. Trait mindfulness is negatively associated with rejection sensitivity (Sheng et al., 2022). In particular, the nonjudgment factor of trait mindfulness negatively predicts rejection sensitivity (Peters et al., 2016). Keng and Tan (2018) demonstrated that brief mindfulness training is effective in improving negative affect and feelings of rejection after social rejection. Furthermore, mindfulness training is effective in improving rumination, social anxiety symptoms, and depressive symptoms (Deyo et al., 2009; Goldin and Gross, 2010). These findings suggest that mindfulness training may be an effective intervention for ADA.

This study has several limitations. First, a longitudinal design should be used to assess the associations among the variables addressed in this study to validate a causal relationship. In the model that we constructed, the direction of causality was assumed based on the results of previous studies, and its validity was examined. However, a cross-sectional design was used in this study; thus, the causal relationships among these variables could not be established. Second, this study was conducted in a specialized clinic for anxiety and depression. Because most patients visiting this clinic present with anxiety and depressive disorders, the possibility exists that the results may have been biased. Future studies should be conducted in hospitals and clinics that specialize in a wide range of mental illnesses. Third, patients who visited the clinic for the first time were included in this study, and the eligibility criteria included patients treated with and without pharmacotherapy. The model should be assessed separately for patients treated with pharmacotherapy and patients who were not treated with pharmacotherapy. Finally, the development of ADA should be further tested. Adverse childhood experiences and childhood emotional abuse/maltreatment are associated with rejection sensitivity, rumination, and clinical symptoms (Chesin et al., 2015; Pierce et al., 2018; Mansueto et al., 2021). A model showing the effects of rejection sensitivity on ADA could be established in the future.

## Conclusion

This study provides preliminary evidence suggesting that rumination, social anxiety symptoms, and depressive symptoms mediate the effect of rejection sensitivity on the development of ADA. This evidence is an impetus for future research to understand the psychopathogenesis of ADA and its treatment.

## Data availability statement

Detailed data are available from the corresponding author upon reasonable request.

## Ethics statement

The studies involving human participants were reviewed and approved by Medical Corporation Warakukai Ethics Review Board. The patients/participants provided their written informed consent to participate in this study.

## Author contributions

HK organized the study. MM, TK, and HK collected the data. SN and HK designed the methods and wrote the first draft of the manuscript. All authors contributed to the article and approved the submitted version.

## Conflict of interest

The authors declare that the research was conducted in the absence of any commercial or financial relationships that could be construed as a potential conflict of interest.

## Publisher’s note

All claims expressed in this article are solely those of the authors and do not necessarily represent those of their affiliated organizations, or those of the publisher, the editors and the reviewers. Any product that may be evaluated in this article, or claim that may be made by its manufacturer, is not guaranteed or endorsed by the publisher.
